# Shapeshifting molecules: the story so far and the shape of things to come

**DOI:** 10.1039/c9sc05482k

**Published:** 2019-12-05

**Authors:** Aisha N. Bismillah, Brette M. Chapin, Burhan A. Hussein, Paul R. McGonigal

**Affiliations:** a Department of Chemistry , Durham University , Lower Mountjoy, Stockton Road , Durham , DH1 3LE , UK . Email: paul.mcgonigal@durham.ac.uk

## Abstract

Shapeshifting molecules exhibit rapid constitutional dynamics while remaining stable, isolable molecules, making them promising artificial scaffolds from which to explore complex biological systems and create new functional materials. However, their structural complexity presents challenges for designing their syntheses and understanding their equilibria. This minireview showcases (1) recent applications of highly dynamic shapeshifting molecules in sensing and distinguishing complex small molecules and (2) detailed insights into the adaptation of tractable bistable systems to changes in their local environment. The current status of this field is summarised and its future prospects are discussed.

## Abstract 2

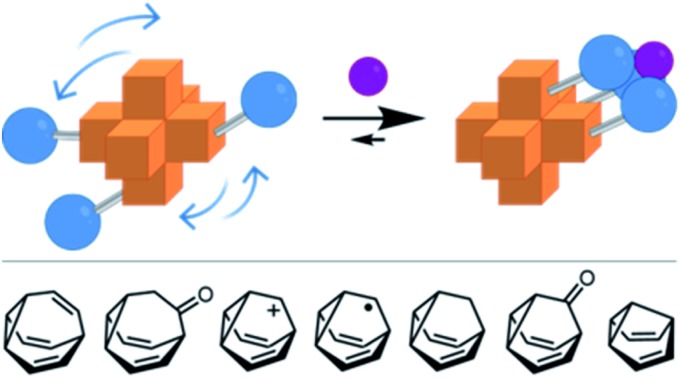
This review describes the synthesis and application of shapeshifting molecules, whose covalent skeletons dynamically reconfigure in response to their surroundings.

## Introduction

The carbon–carbon bonds that hold together organic molecules are generally static, being localised at a fixed in position in a molecular structure. Of course, this characteristic is often desirable. It imparts stability, allowing us to routinely and predictably synthesise isolable new structures based on carbon skeletons. But such a fixed covalent bonding constitution inevitably imposes some restrictions on the three-dimensional shapes accessible to a molecule, particularly for cyclic systems with their well-defined conformational degrees of freedom. The relatively fixed shape, in turn, dictates physical properties and limits noncovalent bonding modes with the surrounding environment.

**Fig. 1:**
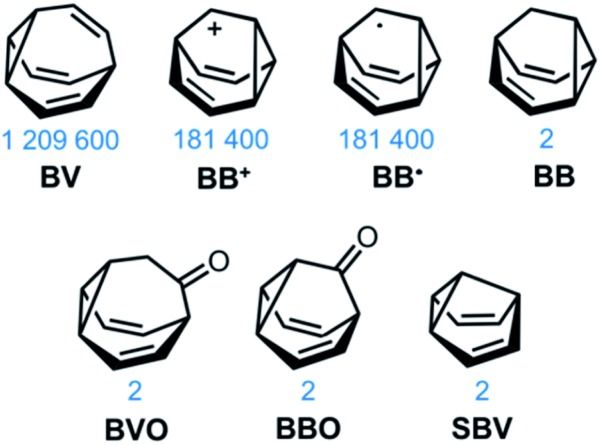
Structural formulas of fluxional carbon cages **BV**, **BB^+^**, **BB˙**, **BB**, **BVO**, **BBO**, and **SBV**. The number of degenerate isomers they each access is given below the structures.

This minireview deals with compounds (Fig. 1) that are exceptions to these general rules. The fluxional tricyclic hydrocarbon cages bullvalene^1^**BV**, the barbaralyl cation^2^**BB^+^**, the barbaralyl radical^3^**BB˙**, barbaralane^4^**BB**, bullvalone^5^**BVO**, barbaralone^5^**BBO** and semibullvalene^6^**SBV** all undergo rapid and reversible pericyclic rearrangements, allowing their atomic skeletons to reconfigure dynamically without breaking apart.

**Scheme 1:**
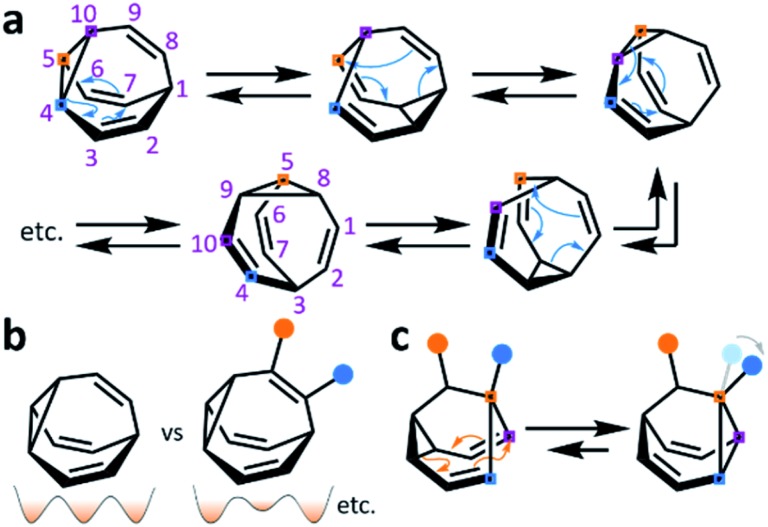
(a) **BV** and five of its valence isomers accessed through Cope rearrangements, where coloured squares and numbers show the movement of the cyclopropane carbon atoms from the initial structure. (b) Comparison of **BV** and a functionalised derivative, which can isomerise between nondegenerate regioisomers. A schematic representation illustrates changes in the degeneracy of the potential energy surfaces. (c) A substituted barbaralane and its Cope rearrangement demonstrating dynamic constitutional isomerism, where coloured squares illustrate carbon atoms remaining in the same sequence in space. Blue and orange circles represent different functional groups.

While some of these fluxional cages are bistable, *i.e.*, they switch between just two states, others access hundreds of thousands of isomers. **BV**, for example, exists as a mixture of 1 209 600 degenerate valence isomers. Each of its methine vertices trades places (Scheme 1a) with every other methine group through a series of Cope rearrangements, leading to 10!/3 discrete structural permutations.

Functionalised derivatives of the fluxional cages take on rather unusual ‘shapeshifting’ properties. Conceptually, by substituting (Scheme 1b) one or more of the hydrogen atoms of the parent structure with another group, the degeneracy of the system is reduced. The dynamic pericyclic rearrangements cause (Scheme 1c) the relative positions and orientations of any appended functional groups to switch back and forth, producing distinct molecular shapes. These shapeshifting molecules, therefore, exist as fluxional mixtures that can be considered as self-contained dynamic structural libraries. Recent advances in synthetic methods^7^–^9^ combined with resourceful computational calculations^9^ have made it possible to investigate the application of these of highly dynamic systems as sensing molecules for structurally complex analytes.^7^ Investigations involving tractable bistable systems have also shed light on how the shapeshifting equilibria adapt in response to external changes.^10^ This minireview summarises these recent advances and discusses areas for future development.

## Dynamic pericyclic rearrangements

Reversible pericyclic processes underpin the constitutional dynamics of fluxional carbon cages. Neutral, closed-shell species such as **BV**, **BB**, **BVO**, **BBO**, **SBV** and their derivatives undergo strain-assisted Cope rearrangements – [3,3]-sigmatropic rearrangements whereby a σ-bond of the cyclopropyl group, flanked on two sides by vinyl π-electron systems, migrates (Scheme 1a) along the π-system with a concerted redistribution of the two π-bonds. The cationic species **BB^+^** undergoes a phenomenologically similar redistribution of bonding electrons between a cyclopropyl σ-bond, a vinyl π-bond, and the vacant p-orbital, whereas the **BB˙** radical rearranges through a series of β-scission and cyclisation steps to open and close the cyclopropyl ring.^3^

**BB**, **BVO**, **BBO** and **SBV** each possess just two olefin ‘arms’ connected to their cyclopropane ring. Consequently, there is only ever one pathway for a Cope rearrangement to follow, which reorganises the bonding electrons but does not change (Scheme 1c) the sequence of methine groups in three-dimensional space. **BB**, **BVO**, **BBO**, **SBV** and their derivatives, therefore, fluctuate between just two isomers. **BV**, however, possesses three olefins, rendering all three ‘arms’ of the carbon cage available to participate in sigmatropic rearrangements. As a series of sequential rearrangements progresses, there are divergent Cope pathways available at each step, involving two olefin arms at a time. Consequently, by proceeding along a pathway that involves (Scheme 1a) each olefin pair in turn, methine vertices that neighbour one another initially are shifted to different positions on opposing sides of the molecular structure. This causes complete ‘scrambling’ of the groups around the **BV** core and leads to the vast number of accessible constitutional isomers.^1^ In an analogous manner, the cationic rearrangements of **BB^+^** involve either one of the two olefins groups at each step, along with the cyclopropane and the carbenium centre.2*b* Similarly, the rearrangements of **BB˙** involve the methine-centred radical, the cyclopropane, and one of the olefin arms. Complete scrambling of the methine groups of **BB^+^** and **BB˙** by participation of all three arms of the cage in the stepwise rearrangement leads to a dynamic mixture of 9!/2 (181 400) degenerate isomers.

The activation energies for the Cope rearrangements of **BV**, **BB**, **BVO**, **BBO**, and **SBV** are atypically low (∼30–50 kJ mol^–1^) when compared to Cope rearrangements of acyclic systems (∼150 kJ mol^–1^).^11^ These low activation energy barriers arise because of (1) the ring strain of the cyclopropyl groups, which destabilises the ground states, and (2) the boat-like conformations enforced by the tricyclic systems, which resemble the transition-state geometries.1*a* The cationic rearrangements of **BB^+^** proceed through two different transition states with activation energy barriers of ≤16 and 21 kJ mol^–1^, respectively.2*b*

Overall, the low energy barriers associated with these rearrangements causes isomers to interconvert extremely rapidly. The constitutional dynamics of the fluxional cages occur at rates more commonly associated with conformational dynamics, *e.g.*, a cyclohexane chair flip proceeds with an energy barrier of 45 kJ mol^–1^. Generally, when observed at room temperature by ^1^H NMR spectroscopy, the rapid interconversion of constitutional isomers results in exchanging nuclei appearing equivalent. For example, at room temperature the ^1^H NMR spectrum of **BV** exhibits just one broad proton resonance, which becomes a single sharp signal at higher temperatures.1*c* The chemical shift of the resonance is a weighted average of the four distinct proton environments present in **BV**.

## Complex fluxional milieux

### Early syntheses of bullvalene and its derivatives

With their large number of possible isomers, **BV** derivatives give access to complex dynamic mixtures. The structure and fluxional properties of **BV** were predicted in 1963 by von Doering.1*a* But, before the dynamics could be explored, new synthetic methods were required to prepare **BV**.

**Scheme 2:**
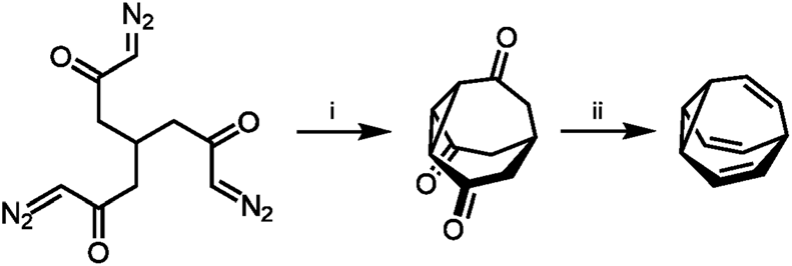
Serratosa's stepwise **BV** synthesis.^12^ Reagents and conditions: (i) CuSO_4_, PhSMe, xylene, 140 °C, ∼2%; (ii) (1) TsNHNH_2_, (2) MeLi, 20%.

The first breakthrough came almost immediately, when Schröder reported the serendipitous discovery of **BV** upon photolysis of a cyclooctatetraene dimer.1*b* Inspired by this first synthetic procedure, several synthetic routes to **BV** were developed.^5^,^12^–^16^ Some methodologies follow Schröder's precedent by employing photochemical transformations of bicyclic hydrocarbon precursors to form **BV**. von Doering^17^ and Scott^18^ showed that photoisomerisation of 4*a*,8*a*-dihydronaphthalene gives access to **BV** with improved yields. Others built up the tricyclic ring system from acyclic or monocyclic precursors. Serratosa^12^ (Scheme 2) and von Doering^5^ each developed stepwise pathways to construct the **BV** skeleton, using ketone intermediates as functional handles to later introduce olefin groups. Subsequently, the preparation of various mono- and oligosubstituted **BV** derivatives were pioneered by Schröder, Oth *et al.* who devised conditions to brominate **BV**, giving bromobullvalene^13^ and dibromobullvalene,^14^ which were used as common intermediates for further elaboration.^15^,^16^ These initial series of compounds allowed (1) the fully degenerate Cope rearrangements of **BV** and (2) the nondegenerate rearrangements of simple derivatives to be observed experimentally.

Below, we discuss the recent synthetic advances that have led to more streamlined, mild syntheses of **BV** derivatives, as well as quantum computational analyses that predict the energetics of the resulting isomer networks.^8^,^9^

### Recent advances in bullvalene synthesis and analysis

**Scheme 3:**
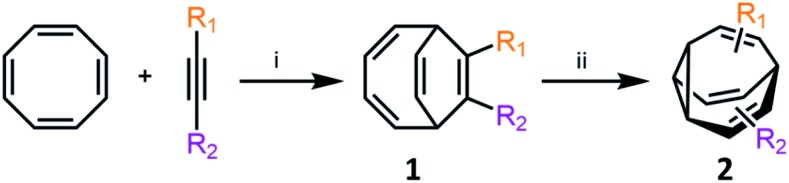
Fallon's concise synthesis of **BV** (R_1_ = R_2_ = H) and a variety of mono- and disubstituted analogues **2**.^9^ Reagents and conditions: (i) CoBr_2_(dppe) (10 mol%), ZnI_2_ (20 mol%), Zn dust (30 mol%), 1,2-dichloroethane, 25 °C or 55 °C, 16–24 h, 63–100%; (ii) *hν*, Me_2_CO, 25 °C, 16–24 h, 35–81%.

Metal-catalysed cyclisation approaches have recently expanded the scope of easily accessible **BV** derivatives. Echavarren^8^ and Fallon^9^ have employed Au(i)-catalysed and Co(i)-catalysed reactions, respectively, as crucial steps to form **BV** precursors. The approach reported by Echavarren *et al.*^8^ proceeds through **BBO**-type intermediates, which are discussed below. Fallon's protocol converts (Scheme 3) cyclooctatetraene into **BV**, or a range of mono- and disubstituted analogues, through an elegant two-step procedure in overall yields of 28–70%.^9^ In the first step, bicyclo[4.2.2]deca-2,4,7,9-tetraene intermediates **1** are prepared by formal [6 + 2] cycloaddition of cyclooctatetraene with alkynes in the presence of a CoI_2_(dppe)/ZnI_2_/Zn catalyst system.^19^ In the second step, **1** undergoes a di-π-methane photoisomerisation^18^ under UV irradiation to give the **BV** derivative **2**. The product bears up to two functional groups that are introduced as part of the alkyne precursor during the first reaction step. While the photoisomerisation procedure tolerates hydroxymethyl, alkyl, benzyl and silyl groups, proceeding in 40–81% yields, aryl and electron-deficient alkyne derivatives are not yet compatible. Nevertheless, the protocol remains the most concise and efficient synthesis of functionalised **BV** derivatives reported to date. While this article was under review, Fallon reported the successful application of this protocol to prepare trisubstituted **BV** derivatives.^20^ Trisubstituted analogues of **1** are prepared in overall yields of 24–50% using trimethylsilylcyclooctatetraene in place of cyclooctatetraene.

**Fig. 2:**
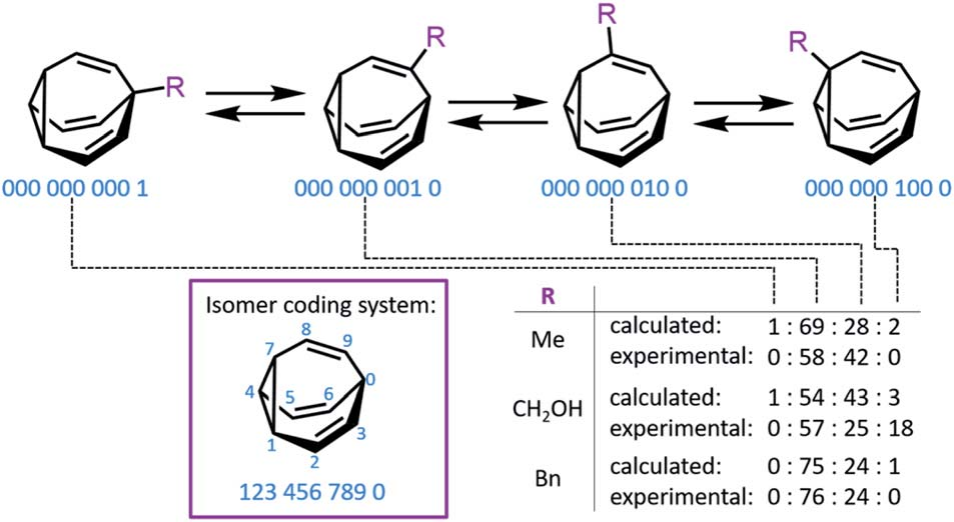
An isomer coding system for network analysis and the computationally predicted isomeric ratios for three monosubstituted **BV** derivatives.

Bode developed a convenient isomer coding system for the network analysis of **BV** derivatives and their substitution patterns.7*f* A ten-digit code is assigned to each possible isomer, where each digit represents (Fig. 2) a position of the **BV** core and the number designates the type of substituent (*e.g.*, using 0 for hydrogen, *etc.*). Fallon *et al.* built upon this isomer coding system for the network analysis of **BV** derivatives by developing an algorithm that integrates it with quantum-chemical energy calculations.^9^,^20^ The algorithm generates all the possible **BV** structures in the dynamic network, identifying enantiomeric pairs and interconnections (*i.e.*, transition states^11^) between isomers. The geometry of each isomer is then optimised and their single-point energies are calculated through the ORCA 4.0 programme package,^21^ forecasting the equilibrium distribution of isomers. Comparison (Fig. 2) of the experimental isomer ratios for mono-, di- and trisubstituted **BV** derivatives (determined by low-temperature NMR) with the modelled ratios illustrated the method's potential to function as a predictive tool. This algorithm and other similar computational techniques will become increasingly valuable if complex **BV** milieux that respond to external stimuli are to be exploited routinely, for example, as chemical sensors, like those described below.

### Applications of shapeshifting mixtures

Early attempts to use functionalised **BV** derivatives as adaptive shapeshifting mixtures were reported by Schröder and co-workers.^22^ They synthesised crown ether-type compounds with **BV** linkages as part of their macrocyclic backbones, aiming to investigate the compounds' association with metal-ion guests. The macrocyclic ring sizes fluctuate back and forth as the dynamic Cope rearrangements proceed. However, in practice, the binding affinities of Schröder's crown ethers for metal ions are very low. Consequently, it was not possible to establish if the equilibrium distribution of macrocycle sizes adapts to match a particular metal ion.

**Fig. 3:**
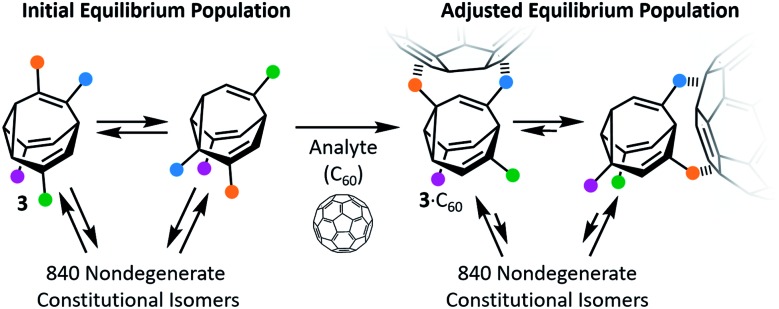
The equilibrium distribution of isomers of shapeshifting molecules **3** are perturbed upon addition of a guest (illustrated with C_60_), favouring structures that form the most stable supramolecular complexes. Coloured dots represent pendant groups connected to the **BV** cores.

**Fig. 4:**
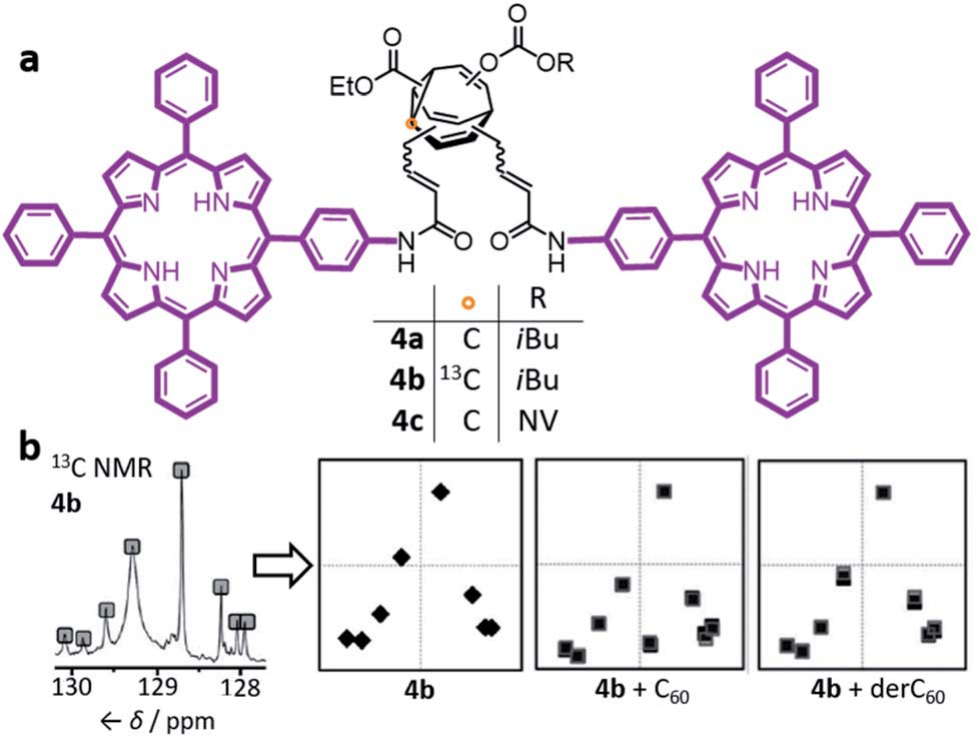
(a) Bode's bisporphyrin-bullvalene **4**, which was prepared with natural isotopic abundance (**4a**), enriched in ^13^C at one position (**4b**) of the dynamic C_10_ core, and functionalised with a photocleavable group (**4c**). (b) The complex ^13^C NMR spectra of the dynamic mixtures are converted to simplified representations, showing that the ^13^C NMR spectrum of a **4b** mixture at equilibrium changes in the presence of fullerene guests. NMR data (150 MHz, solutions in CS_2_ with 3% CD_2_Cl_2_) shown for samples of: (**4b**) 2.8 mM **4b**; (**4b** + C_60_) 2.8 mM **4b** with 2 equiv. C_60_; (**4b** + derC_60_) 2.8 mM **4b** with 4 equiv. derC_60_. The representations of NMR spectra correspond to two trials overlaid. NV = *o*-nitroveratryl. Adapted from ref. 7*e* with permission from The Royal Society of Chemistry.

In the past decade, however, Bode *et al.* have successfully demonstrated^7^ responsive networks of fluxional isomers **3**. The equilibrium mixtures of **3** preferentially adopt structures that interact favourably (Fig. 3) with guests. They synthesised functionalised tetrasubstituted **BV** derivatives **4** following a multistep route from cycloheptanone.7a,c A late-stage olefin metathesis introduced (Fig. 4) two porphyrin groups to the **BV** core through acrylamide linkers.7b,e A series of analogues have been prepared from precursors with (1) natural isotopic abundance (**4a**), (2) an atom of the **BV** core enriched in ^13^C (**4b**) to aid NMR analysis, or (3) the inclusion of a *o*-nitroveratryl (NV) carbonate function (**4c**) as a photocleavable group.

The porphyrin groups present in **4** have high affinities for C_60_ by virtue of aromatic interactions. Bode compared the binding affinities of C_60_ towards bisporphyrin **4a** and an analogous bistable bisporphyrin **BVO** derivative.7*b*^1^H NMR spectroscopic titrations revealed that the fully dynamic compound **4a** has an association constant, *K*_a_, of 920 ± 100 M^–1^ for C_60_ in C_6_D_5_CD_3_, which can be compared to the *K*_a_ of 570 ± 65 M^–1^ measured for its **BVO** analogue. The higher *K*_a_ of **4a** can be attributed to adaptation of its dynamic mixture of constitutional isomers, favouring structures that form the most stable supramolecular complexes with C_60_. This adaptation has been visualised by monitoring (Fig. 4b) changes in the ^13^C NMR spectra of isotopomer **4b**.7*e* The ^13^C NMR spectra of the mixtures are depicted in a simplified manner by plotting the chemical shifts of peaks against their intensities. A characteristic plot is obtained for the **4b**–C_60_ mixture, which can be distinguished from both the **4b** host and from a mixture with a pyrrolidine-derivatised C_60_ fullerene (derC_60_).

By exposing a mixture of **4c** and C_60_ to UV irradiation, Bode was able to ‘trap’ the dynamic library of shapeshifting hosts after it had adapted in response to binding the C_60_ guest.7*c* The NV group of **4c** is lost by homolytic cleavage of the NV–oxygen bond, followed by liberation of CO_2_ from the resulting carbonate radical and transformation of the **BV** core into a bistable **BVO** derivative. The trapped mixture could be analysed and fractionated by HPLC. Bode observed that this adapted **BVO** mixture exhibits an approximately two-fold enhancement in binding for C_60_ when compared to a control compound—a single, synthetically prepared **BVO** derivative that has not been structurally optimised for binding. Overall, the observations made by studying **4** illustrate that the C_60_ guest moulds the covalent structure of the shapeshifting **BV** receptor, skewing the equilibrium distribution of isomers towards those with greater binding affinities.

**Fig. 5:**
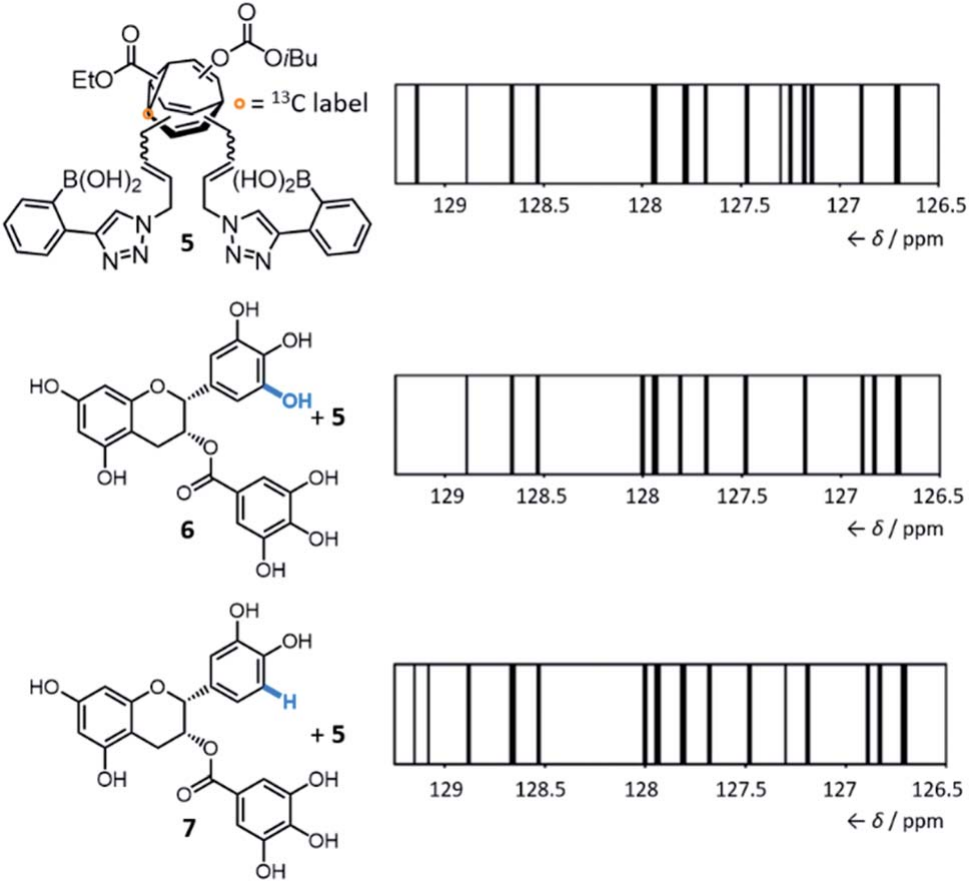
The equilibrium distribution of ^13^C-labelled bisboronic acid **5**, which can be monitored by its NMR ‘barcode’, which changes in a characteristic manner upon interaction with polyols. Even structurally similar polyols **6** and **7** cause distinctive changes in the barcode. The ^13^C NMR resonance of **6** at 129.1 ppm and of **7** at 129.8 ppm are omitted from the respective barcodes. Conditions: 2.5 μmol **5** and 5.0 μmol of the corresponding analyte were dissolved in 0.6 mL of a 9 : 1 mixture of (CD_3_)_2_SO/phosphate buffer (0.05 M, pH = 7.2), followed by equilibration for 1 h at rt and analysis by ^13^C NMR (typically 14 500 scans). Adapted with permission from J. F. Teichert, D. Mazunin and J. W. Bode, *J. Am. Chem. Soc.*, 2013, **135**, 11314. Copyright 2013 American Chemical Society.

By introducing (Fig. 5) boronic acid end groups, Bode *et al.* prepared a **BV** derivative, **5**, that is set up to interact with and sense polyols through dynamic covalent condensation reactions, forming cyclic boronic ester linkages.7*g* They allowed shapeshifting mixtures of **5** to condense with a series of structurally complex polyol analytes, using ^13^C NMR analysis of the isotopically labelled **BV** as a non-destructive readout. The targets included small biomolecules such as sucrose, fructose, sialic acids, epigallocatechin gallate **6** and epicatechin gallate **7**. Selective sensing of these compounds is challenging because of (1) their overall structural complexity (bearing several stereo-centres and multiple hydroxyl groups) and (2) their similarity to one another. For example, **6** and **7** differ (Fig. 5) by only one hydroxyl group. Impressively, they demonstrated that the ^13^C NMR peak pattern of **5** is distinctive and reproducible in the presence of each of the different polyols. For ease of comparison and to reduce the complexity of the spectra, the chemical shifts and peak heights were extracted for each ^13^C NMR spectrum of the **BV** derivative and the signals were displayed as ‘barcodes’.^23^ As a negative control, a structurally similar non-dynamic bis-boronic acid was synthesised and tested as a sensor. Its lack of fluxionality led to only one set of ^13^C signals being observed with each analyte, preventing it from being used to identify each analyte based on the ^13^C peak pattern. The response of **5** to mixtures of polyol analytes was also tested, revealing that the strongest binding analyte dominates the corresponding barcode.

### Prospects for the applications of shapeshifting sensors

Currently, there are several open challenges and opportunities for the concept and applications of these shapeshifting sensors. Their remarkable ability to differentiate (Fig. 5) very similar analytes from one another is a particularly promising characteristic. However, there is scope to expand and improve upon the technique used as a readout. The ^13^C NMR spectroscopic methods developed by Bode are data rich and well resolved, which are necessary characteristics to effectively distinguish complex guests. But at present, the differences in the ^13^C NMR barcodes are subtle. Advances in the molecular design of the sensors may improve differentiation between analytes – for example, the flexible acrylamide linkers of **4** and **5** introduce a high degree of conformational flexibility, which presumably diminishes the geometric differences between the **BV** isomers. Systems with shorter, more rigid functional handles would likely show improved performance. Future developments may also focus on using complementary spectroscopic techniques. Perhaps more readily available instrumentation and low-cost methods can be applied (*e.g.*, infrared or fluorescence spectroscopy), although there would likely be a trade-off in the resolving power of the data obtained. Care will need to be taken when designing systems to minimise the inherent Gibbs free energy differences between isomers. The introduction of substituents to the **BV** core has been found7f,^9^,^20^ to favour a small number of isomers by energy differences on the order of >40 kJ mol^–1^, which would likely limit the sensitivity of the system to small changes in noncovalent bonding interactions. There is also considerable scope to investigate the use of alternative end groups, diverging from the boronic acid and porphyrin chemistry employed to date. Ultimately, such advances may make shapeshifting **BV** systems appealing candidates for sensing and binding yet more complex targets, such as proteins and other biopolymers.

## Tractable nondegenerate systems

### Established synthetic approaches to barbaralanes

**Scheme 4:**
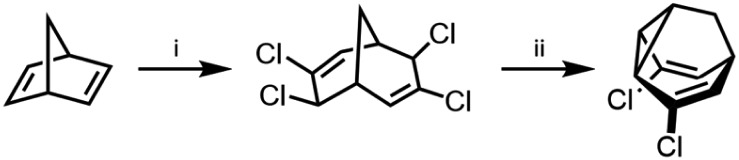
Dihalogenated **BB** synthesis from norbornadiene.^27^ Reagents and conditions: (i) CHCl_3_, Cetrimide®, NaOH, H_2_O, 30 °C, 2 h, 18%; (ii) Zn, Me_2_CO, 56 °C, 6.5 h, 90%.

Compared to the complexity of the **BV** equilibria, the relative simplicity of bistable **BB** systems makes them attractive targets for developing a quantitative understanding of shapeshifting equilibria. **BBO** was first prepared as part of von Doering's investigations into thermal rearrangements that led to the **BV** structure being proposed.^24^ The original strategy, which has been optimised by others,^25^ was to employ an intramolecular [2 + 1] cycloaddition of a carbene to a cycloheptatriene to form the tricyclic **BB** core. Subsequently, methods have been developed that avoid the low-yielding cyclisation of a carbenoid intermediate. Henkel reported a five-step synthesis of **BB** from 2-adamantanone in an overall yield of 42%, proceeding by fragmentation of the adamantane system followed by a transannular ring closure.^26^ Efficient syntheses of substituted **BB** derivatives have been developed that employ norbornadiene^27^ (Scheme 4) or Meerwein's diketone^28^,^29^ as starting materials. In both cases, the tricyclic **BB** skeleton is accessed by reductive cyclisation of a bicyclic precursor. These multistep routes have proven sufficiently effective to give researchers access to a series of simple **BB** derivatives. Quast *et al.*^29^ quantified the equilibria and rearrangements rates for disubstituted **BB** derivatives,29*c* demonstrating that judicious functionalisation of the vinyl groups with electron-donating or electron-withdrawing substituents tunes the barrier to Cope rearrangement over a wide range from <17.2 to 44.9 kJ mol^–1^.

### Recent advances in barbaralane synthesis

**Scheme 5:**
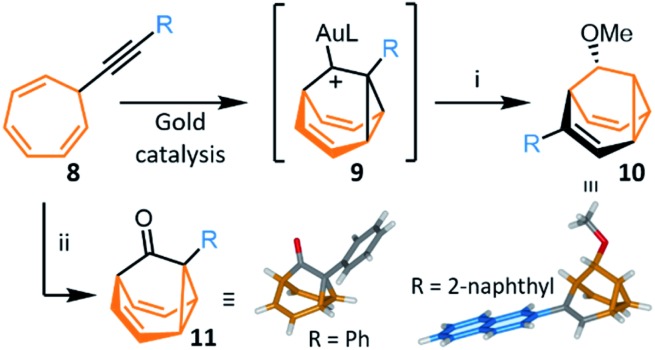
Gold-catalysed transformation of alkynyl cycloheptatrienes **8** into a barbaralyl methyl ether **10** or barbaralones **11**.^8^ X-ray crystal structures of each product are shown. Reagents and conditions: (i) **8**, R = 2-naphthyl, [JohnPhosAu(MeCN)][SbF_6_] (5 mol%), CH_2_Cl_2_–MeOH (1 : 1), rt, 2 h, 40%; (ii) **8**, [IPrAu(MeCN)][SbF_6_] (5 mol%), Ph_2_SO, CH_2_Cl_2_, 1.5–12 h, rt, 35–97%. L = ancillary ligand, JohnPhos = (2-biphenyl)di-*tert*-butylphosphine, IPr = 1,3-bis(2,6-diisopropylphenyl)imidazol-2-ylidene.

The most recent and concise syntheses of **BB** derivatives have been reported by Echavarren *et al.*,^8^ exploiting (Scheme 5) the ability of gold catalysts to activate enynes towards cycloisomerisation. Gold complexes promote a wide variety of enyne cyclisations under mild conditions while exerting exquisite control over competitive reaction pathways, often stabilising intermediates with carbenoid character.^30^ Alkynyl cycloheptatrienes **8** are first prepared by nucleophilic addition of an acetylide to the commercially available reagent^31^ tropylium tetrafluoroborate. Activated (η^2^-alkynyl)gold(i) complexes, formed from **8** in the presence of gold catalysts, cycloisomerise to gold-stabilised fluxional barbaralyl cations **9**. In the absence of nucleophiles, these reactive intermediates transform irreversibly to indenes.8*a* The **BB** framework can be trapped, however, if **9** is intercepted by a nucleophile. By carrying out the cycloisomerisation using methanol as a cosolvent, Echavarren demonstrated that a barbaralane methyl ether **10** can be isolated (Scheme 5) as the major project, alongside a small amount of indenyl by-products. This short two-step pathway gives access to disubstituted **BB** derivatives in which one substituent has been introduced as part of the acetylide and the other is controlled through the choice of nucleophile.

In 2016, Echavarren reported that **BBO** products functionalised at the 1-position, **11**, are formed selectively (Scheme 5) by including an oxidant in the reaction mixture.8*b* The optimised conditions employ diphenyl sulfoxide as oxidant in combination with a gold(i) catalyst bearing a *N*-heterocyclic carbene ancillary ligand, [IPrAu(MeCN)][SbF_6_]. Two mechanisms have been proposed for the transformation. Either the oxidant reacts with the previously observed8*a* intermediate **9**, or the gold catalyst mediates oxidation of the alkyne to afford an α-oxo gold(i) carbene intermediate, which then undergoes intramolecular cyclopropanation to produce **11**. The method was shown to be compatible with a series of alkynyl derivatives **8**, tolerating alkyl groups and electron-rich or electron-poor aromatic substituents bearing *ortho*-, *meta*- or *para*-groups. The isolated yields reported range from 45–97%. This synthesis of **BBO** derivatives is the shortest to date and provides straightforward access to substituted bistable fluxional carbon cages. The parent **BBO**, which is accessed in 97% from ethynyl cycloheptatriene, was subsequently transformed into **BV** in 44% by homologation to **BVO** with the lithium anion of (trimethyl-silyl)diazomethane, before formation of the corresponding enol triflate and reduction with *n*Bu_3_SnH.

### Adaptation of simple nondegenerate mixtures

Investigations during the 1980s and 1990s showed that the dynamics of some shapeshifting molecules are suppressed^32^ in the crystal state, while others remain^33^ highly fluxional. Generally, the compounds that remain dynamic have globular structures, *e.g.*, **BV**33*d* and some of its monohalogenated33e–g derivatives. In these cases, the Cope rearrangements are not associated with a significant change in the three-dimensional shape of the molecules, so the rearrangements can occur within the restricted environment of a crystal lattice. Less regularly shaped compounds, however, such as substituted **BB** derivatives, appear to be frozen in the solid state.32*c* Their valence isomerism leads (Scheme 1c) to a significant shape change that is mismatched with the restricted environment of the crystal lattice.

**Fig. 6:**
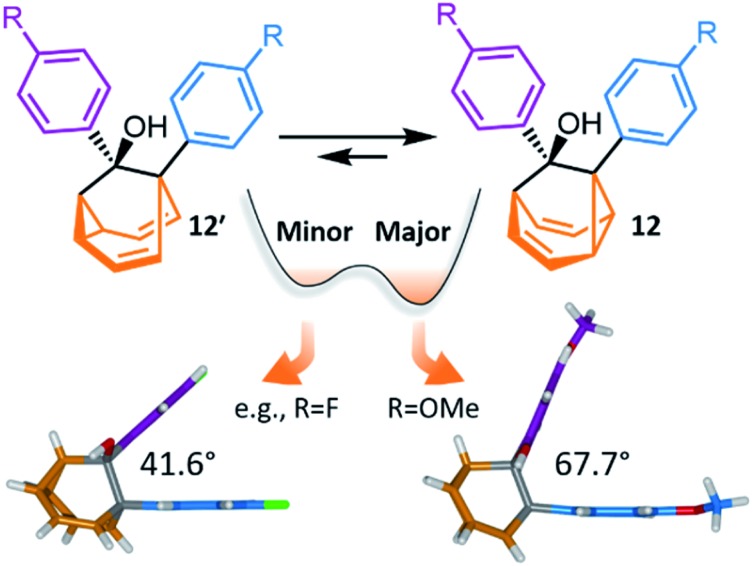
McGonigal reported a series of disubstituted **BB** derivatives, **12**/**12′**, that crystallise in a manner controlled by their shapes, preferring valence isomers that pack effectively in a crystal lattice.^10^ In each case, the same isomer **12** is present as the major species in solution, but small changes in the aromatic substituent, R, favour different solid-state structures. In some cases, the minor solution-state valence isomer is the one observed in the solid state.

Recently, McGonigal has taken advantage of this phenomenon to quantitatively analyse the adaptation of simple nondegenerate **BB** mixtures upon changes (Fig. 6) in their environment.^10^ The investigation started by preparing a series of **BB** derivatives that are bistable, fluctuating between structures **12** and **12′**. Precursors **11** were synthesised (Scheme 5) according to the gold catalysis method described above, before aryl Grignard reagents were added to afford **12**/**12′**. Each compound in the series possesses a tertiary alcohol and aromatic groups at the 1- and 9-positions. The aromatic groups were varied between *p*-anisyl, *p*-fluoro-phenyl and phenyl across the series. NMR analyses confirmed that these **BB** derivatives interconvert between two constitutional isomers, **12** and **12′**, in solution. Isomer **12** is the preferred solution-state isomer in every case and relatively small Gibbs free energy differences between isomers of ∼3–5 kJ mol^–1^ were estimated based on low-temperature NMR data and DFT calculations, suggesting that the positions of the **BB** equilibria would be sensitive to changes in the surrounding medium.

Indeed, upon transitioning from solution to the solid state, the five compounds are no longer isostructural. Three of the five **BB** derivatives were found (Fig. 6) to have the same constitution as their major solution-phase isomer **12** while two were found to take up the structure of the minor solution-phase isomer **12′**. In each case, only one of the two possible valence isomers was observed in the solid state. Variable-temperature X-ray analysis showed that the fluxional dynamics were suppressed.

The simplicity of this bistable system and the relative lack of conformational freedom present in the substrates made it possible to analyse the reasons behind this behaviour in detail. Hirshfeld surfaces of each crystal structure were modelled and the interaction energies between neighbouring molecules in the crystals were calculated. The calculations revealed that, despite the presence of an alcohol group and mildly polarised aromatic rings, the crystal packing is not dictated by any significant and specific noncovalent bonding interactions. Instead, van der Waals interactions constitute the dominant contacts. Since the relatively small energy differences between valence isomers can be outweighed by differences in lattice enthalpies, it becomes possible for the preference for the shape of **12** or **12′** to bias crystallisation. Isomerisation is associated (Fig. 6) with changes in the dihedral angles between the aromatic groups, altering the crystal packing motif. Ultimately, these insights confirm the idea that finely balanced shapeshifting equilibria can adapt in response to changes in supramolecular self-assembly and the molecules' interactions with their surrounding medium. By gaining understanding of the dynamics, equilibria and energetics of bistable shapeshifting molecules, it will be possible to better rationalise and predict the adaptation of shapeshifting systems with more permutations operating in more complex environments.

## Conclusions and outlook

Since the conception of fluxional carbon cages as a thought experiment by von Doering several decades ago, our understanding of them has progressed considerably. Early synthetic approaches gave access to functionalised systems, allowing the first shapeshifting mixtures to be analysed. Advanced **BV** derivatives prepared by multistep syntheses in the past decade have illustrated their potential to act as highly specific sensing molecules, capable of differentiating structurally similar biomolecules from one another. The vast numbers of permutations accessed by shapeshifting molecules offer both opportunities and challenges. On one hand, they are uniquely complex single-molecule species that can be considered as self-contained adaptive systems. The equilibrium distributions of their covalent structures are perturbed in response to changes in their environment, including host–guest interactions with analytes. Unlike dynamic covalent libraries based on intermolecular assembly of building blocks,^34^ the equilibrium populations of fluxional carbons cages are insensitive to changes in concentration, which makes them appealing for applications in dilute environments, such as those found in biological systems or at interfaces. On the other hand, analysis of such complex, rapidly exchanging mixtures is inherently difficult. So far, NMR spectroscopy has been used for the most fine-grained analyses of shapeshifting mixtures, but it is a laborious task to correlate spectral signals to individual structures and to deconvolute equilibria. Alternatively, spectra can be treated as ‘fingerprints’, but doing so cedes the insights available if the changes in equilibria are fully understood. Solutions to this dichotomy are already starting to emerge as computational modelling of the equilibria advances to the level that it can aid in interpreting complex data sets, as well as the discovery of fluxional characteristics in new synthetic systems.^35^ As yet, the influence of shapeshifting rearrangements on materials physical properties has not been investigated in depth. It is tempting to imagine that such large-scale dynamic covalent rearrangements would impart unusual responsive properties if fluxional carbon cages are incorporated, for example, as part of the structures of polymers or liquid crystals. The other major challenge in the area has been the time-consuming and low-yielding syntheses required to access functional shapeshifting systems. However, the recent synthetic advances highlighted here have made **BV** and **BB** derivatives more easily accessible than ever before. More routine access to shapeshifting systems will allow their properties and applications to be explored beyond the areas of synthetic and physical organic chemistry at the interfaces with materials chemistry and biology.

## Conflicts of interest

There are no conflicts to declare.
